# Isolated calf deep venous thrombosis: frequency on venous ultrasound and clinical characteristics

**DOI:** 10.1186/s12873-021-00516-1

**Published:** 2021-10-30

**Authors:** Thomas Heller, Mattes Becher, Jens-Christian Kröger, Ebba Beller, Susanne Heller, Raimund Höft, Marc-André Weber, Felix G. Meinel

**Affiliations:** 1 Institute of Diagnostic and Interventional Radiology, Pediatric Radiology and Neuroradiology, University Medical Centre Rostock, Ernst-Heydemann-Str. 6, 18057 Rostock, Germany; 2 Department of Emergency Medicine, University Medical Centre Rostock, Rostock, Germany

**Keywords:** Deep venous thrombosis, Duplex ultrasound, Compression ultrasound

## Abstract

**Background:**

It remains controversial whether to include calf veins in the initial ultrasound evaluation of suspected deep venous thrombosis (DVT). We sought to investigate the frequency and clinical characteristics of isolated calf DVT.

**Materials and methods:**

In this retrospective analysis, we investigated a cohort of 596 patients (median age 69 years, 52.3% women) who had been imaged with complete lower extremity venous duplex ultrasound for suspected acute DVT. Radiology reports were analyzed for the presence and localization of DVT. Clinical information was collected from patients’ electronic charts.

**Results:**

DVT was found in 157 patients (26.3%), of which 74 patients (47.1%) had isolated calf DVT. Isolated calf DVTs were located in the posterior tibial veins (22 patients, 29.7%), peroneal veins (41 patients, 55.4%) and muscle veins (19 patients, 25.7%). There were no differences in age or sex between patients with isolated calf DVT and patients with proximal DVT. Isolated calf DVT was more commonly associated with leg pain (52.7% vs. 33.7%, *p* = 0.0234) and less commonly associated with subjective leg swelling (35.1% vs. 55.4%, *p* = 0.0158) and objectively measured difference in leg circumference (23% vs. 39.8%, *p* = 0.0268). D-Dimers were significantly lower in patients with isolated lower leg DVT (median 2.3 vs. 6.8 mg/L, *p* < 0.0001) compared to patients with proximal DVT.

**Conclusions:**

Isolated calf DVT represents approximately half of DVT cases and has different clinical characteristics than proximal DVT.

## Background

Venous ultrasound is the standard imaging test for patients with suspected deep venous thrombosis (DVT). There is, however, great variability in the ultrasound technique performed for suspected DVT between institutions [1, 2] and guidelines [3–8]. In particular, there is disagreement on whether to include the calf veins in the initial ultrasound evaluation of suspected DVT. Some institutions perform ultrasound examinations with a limited range from the groin to the knee - sometimes in the form of two-point or three-point compression ultrasound as the initial test [8–15]. If negative, this is typically followed by a second ultrasound examination within one week to safely exclude DVT [8, 16]..

The clinical importance of isolated calf-vein DVT is incompletely understood with regards to the risks for propagation into the popliteal and femoral vein, pulmonary embolism and the development of a post-thrombotic syndrome. A certain percentage of isolated asymptomatic distal DVTs will extend to the proximal veins if untreated. This rate was previously thought to be on the order of 15–20% [17, 18] but was significantly lower (5%) in the placebo arm of the prospective randomized CACTUS trial [19]. Nevertheless, the benefit of treating patients with isolated calf DVT remains uncertain and controversial [8, 18–25].

In light of this ongoing controversy, we sought to investigate the frequency and clinical characteristics of isolated calf DVT on complete duplex ultrasound examination as the initial test in patients with suspected DVT.

## Material and methods

### Study design, ethical approval, and patient selection

**Fig. 1:**
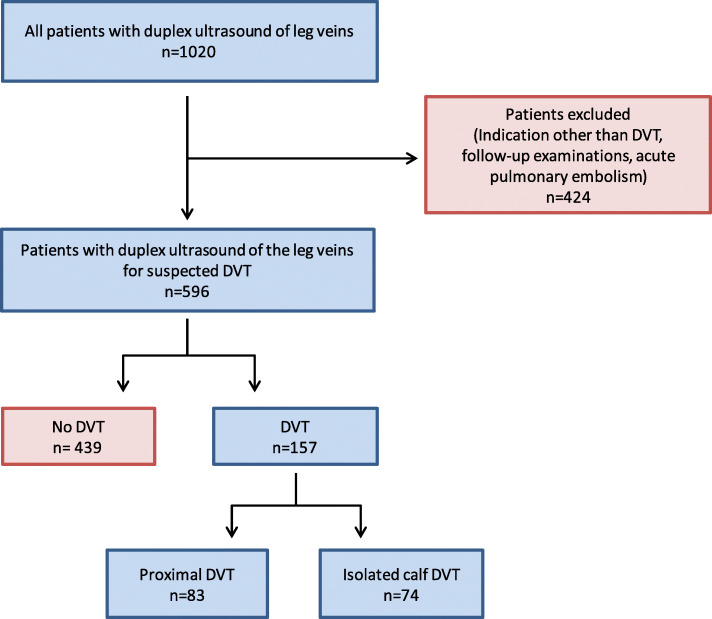
Flow chart of patient inclusion

This study was granted approval by our institutional review board (Ethics Committee, Faculty of Medicine, University of Rostock, Germany). The institutional review board granted permission to access and use the medical records and waived the need for informed consent for this retrospective analysis, in which the collected data was fully anonymized prior to analysis. This was a single-center cohort study. We retrospectively included all patients who were examined with venous ultrasound of the legs at our institution in the year 2014 for suspected acute DVT. During this year, venous ultrasound was predominantly performed by subspecialized radiologists with > 25 years of experience in vascular sonography (TH and JCK). We identified eligible patients through a retrospective search of our radiology information system. We excluded patients in whom venous ultrasound had been performed with an indication other than suspected acute DVT. Follow-up ultrasound examinations in patients with an established diagnosis of DVT in the past 3 months were also excluded from this analysis. (Fig. 1). We further excluded patients referred for duplex ultrasound because of a recent diagnosis of acute pulmonary embolism under the assumption that isolated calf DVT in patients with pulmonary embolism may not be truly isolated calf DVT but rather the remnant of a more extensive proximal DVT, much of which has embolized. We did not exclude patients with a past (> 3 months) history of DVT.

### Ultrasound technique

All ultrasound examinations were performed on a Toshiba Aplio XG SSA 770A ultrasound machine. Based on previous studies demonstrating D-Dimer testing and Wells score are of limited accuracy in isolated calf DVT [26], our practice is to perform venous ultrasound in all patients with clinically suspected DVT irrespective of D-dimer levels and Wells score. Our institutional standard in suspected DVT is to always perform a complete duplex ultrasound of the symptomatic leg(s). Our institution’s technique for whole leg venous ultrasound has been previously described in detail [27].

### Analysis of radiology reports

Radiology reports were retrospectively reviewed by a medical student (initials blinded) for the presence and location of DVT. If the localization could not be precisely determined from the radiology report, the images of the ultrasound examination stored in our PACS were viewed by a board-certified radiologist to determine localization. Patients were classified as having proximal DVT if any portion of the DVT was in the popliteal vein, femoral and/or iliac veins. In contrast, patients were classified as having isolated calf DVT, if DVT was exclusively in one or more veins below the knee (posterior tibial veins, peroneal veins or muscle veins of the calf). Patients with bilateral DVT were classified according to the leg with the more proximally located DVT.

### Analysis of clinical data

Clinical information including age, sex, signs and symptoms, risk factors, Wells scores and D-Dimer levels were collected from patients’ electronic charts.

### Statistical analysis

Commercially available software (GraphPad Prism, version 8.4.2, GraphPad Software Inc) was used for statistical analysis. For continuous variables, 25th, 50th (median) and 75th percentile were calculated. The Mann-Whitney test was used for comparison of continuous variables. For categorical variables, frequencies and proportions were calculated. Fisher’s exact test was used to compare the distribution of categorical variables between groups. An alpha level of <.05 was considered to indicated statistically significant differences.

## Results

### Patient characteristics

Our final study cohort consisted of 596 patients, of which 312 (52.3%) were women. Patient characteristics are summarized in Table 1. Median age was 69 years. The most common local symptoms across all 596 patients were leg pain (40.9%) and leg swelling (40.6%). 12.9% of patients had active malignancy and 16.4% of patients had a past medical history of DVT (Table 1).

**Table 1:** Characteristics of Study Population

		All patients (*n* = 596)		No DVT (*n* = 439)		DVT (*n* = 157)		*P*-Value
		N	%	n	%	N	%	
Females		312	52.3%	223	50.8%	89	56.7%	0.2264
Age in years, median (interquartile range)		69 (55–78)		69 (55–77)		70 (55–79)		0.2393
Presentation	Leg pain	244	40.9%	177	40.3%	67	42.7%	0.6368
	Leg swelling	242	40.6%	170	38.7%	72	45.9%	0.1300
	Circumference difference	161	27%	111	25.3%	50	31.8%	0.1172
	Redness	40	6.7%	30	6.8%	10	6.4%	0.9999
Risk factors	Known coagulopathy	9	1.5%	6	1.4%	3	1.9%	0.7043
	Active cancer	77	12.9%	53	12.1%	24	15.3%	0.3320
	Previous DVT	98	16.4%	52	11.8%	46	29.3%	**0.0004**
	Wells score, median (interquartile range)	1 (0–2) [*n* = 188]		1 (0–2) [*n* = 135]		2 (1–4) [*n* = 53]		**< 0.0001**
Lab	D-dimers in mg/L, median (interquartile range)	1.7 (0.8–4.1) [*n* = 347]		1.4 (0.6–2.8) [*n* = 252]		4.2 (1.9–9.5) [*n* = 95]		**< 0.0001**

*P*-values < 0.05 appear bold

### Comparison of patients with DVT vs. patients without DVT on ultrasound

DVT was found in 157 of 596 patients (26.3%, Table 1 and Fig. 1). DVT was right-sided in 71 of 157 patients (45.2%), and left-sided in 71 of 157 patients (45.2%), resulting in a right/left ratio of 1.0. 15 patients had bilateral DVT (9.6% of all patients with DVT). Patients with DVT were significantly more likely to have a prior history of DVT (29.3% vs. 11.8%, *p* = 0.0004) than patients without DVT on ultrasound. Patients with DVT had higher Wells scores (median 2 vs. 1, *p* < 0.0001) and higher D-Dimer levels (median 4.2 vs. 1.4 mg/L, *p* < 0.0001) than patients without DVT. There were no differences in age, sex or leg symptoms between patients with and without DVT on ultrasound.

### Frequency of isolated lower leg DVT

Among the 157 patients with DVT, 74 patients (47.1%) had isolated lower leg DVT. Isolated calf DVT was right-sided in 34 of 74 patients (45.9%), and left-sided in 38 of 74 patients (51.4%), resulting in a right/left ratio of 0.9. Calf DVT was bilateral in 2 cases (2.7%). Isolated lower leg DVTs were located in the posterior tibial veins in 22 patients (29.7%), peroneal veins in 41 patients (55.4%) and muscle veins (gastrocnemius or soleus) in 19 patients (25.7%). Eight of these patients had DVT in multiple calf veins.

### Comparison of patients with isolated lower leg DVT vs. patients with proximal DVT

There were no differences in age, sex or risk factors between patients with isolated lower leg DVT and patients with a proximal DVT (Table 2). Isolated lower leg DVT was more commonly associated with leg pain (52.7% vs. 33.7%, *p* = 0.0234) and less commonly associated with subjective leg swelling (35.1% vs. 55.4%, *p* = 0.0158) and objectively measured difference in leg circumference (23% vs. 39.8%, *p* = 0.0268). There was a trend for Wells score to be lower in patients with isolated lower leg DVT (median 2 vs. 3, *p* = 0.0915). D-Dimers were significantly lower in patients with isolated lower leg DVT (median 2.3 vs. 6.8 mg/L, *p* < 0.0001) compared to patients with proximal DVT. Three of 74 patients (4.1%) with isolated calf DVT had normal D-dimers (< 0.5 mg/L) compared to 1 of 83 patients (1.2%) with proximal DVT.

**Table 2:** Characteristics of patients with isolated lower leg DVT vs. proximal DVT

		All patients with DVT (*n* = 157)		Proximal DVT (*n* = 83)		Isolated lower leg DVT (*n* = 74)		*P*-Value
		N	%	n	%	n	%	
Females		89	56.7%	45	54.2%	44	59.5%	0.5233
Age in years, median (interquartile range)		70 (55–79)		70 (55–79.5)		69.5 (55–78.75)		0.7689
Presentation	Leg pain	67	42.7%	28	33.7%	39	52.7%	**0.0234**
	Leg swelling	72	45.9%	46	55.4%	26	35.1%	**0.0158**
	Circumference difference	50	31.8%	33	39.8%	17	23%	**0.0268**
	Redness	10	6.4%	7	8.4%	3	4.1%	0.3361
Risk factors	Known coagulopathy	3	1.9%	2	2.4%	1	1.4%	0.9999
	Active cancer	24	15.3%	15	18.1%	9	12.2%	0.3765
	Previous DVT	46	29.3%	25	30.1%	21	28.4%	0.8616
	Wells score, median (interquartile range)	2 (1–4) [*n* = 53]		3 (1–4) [*n* = 25]		2 (1–3) [*n* = 28]		0.0915
Lab	D-Dimer in mg/L, median (interquartile range)	4.2 (1.9–9.5) [*n* = 95]		6.8 (4.2–12) [*n* = 51]		2.3 (1.1–5.0) [*n* = 44]		**< 0.0001**

*P*-values < 0.05 appear bold

## Discussion

Isolated calf vein DVT is frequent and represents 28 to 70% of all lower-limb DVTs diagnosed on ultrasound series [15, 28–33]. Our results are in line with these earlier reports since we found that in our series 47% of DVTs diagnosed on complete duplex ultrasound as the initial tests were isolated calf vein DVTs. This suggests that an initial ultrasound evaluation performed with a limited range (from the groin to the popliteal vein) will miss almost half of DVT cases.

Our study goes beyond previously published data, as we analyzed the clinical characteristics of patients with isolated calf DVT compared to proximal DVT. Interestingly, we observed that isolated calf DVT was more commonly associated with leg pain than proximal DVT. It is generally thought that acute DVT triggers an inflammatory response [34] and that pain from DVT predominantly results from inflammation of the venous wall around the clot. A possible interpretation of our results would be that the local inflammatory response to DVT may be more pronounced in the smaller calf veins that in larger proximal veins.

Less surprisingly, we found that isolated calf DVT was less commonly associated with subjective leg swelling and objectively measured circumference difference. The most straightforward explanation for this finding is that in most patients, there is a single iliac, femoral and popliteal vein for each leg. DVT in these veins will thus occlude the entire deep venous outflow at this level. In contrast, there are multiple deep veins in the calf (typically paired peroneal veins, anterior and posterior tibial veins as well as muscle veins). Most cases of isolated calf DVT will occlude one or few of these veins and leave other deep calf veins patent.

Regarding laboratory values, we observed that levels of D-dimers were significantly lower in patients with isolated lower leg DVT compared to patients with proximal DVT. This likely reflects the greater thrombus burden in patients with DVT in larger, more proximal veins compared to isolated calf DVT. In our analysis, 4.1% of patients with isolated calf DVT had “negative” D-dimers (reference value of our hospital laboratory < 0,5 mg/L) compared to 1 of 83 patients (1.2%) with proximal DVT, suggesting that D-dimer testing is somewhat less sensitive for isolated calf DVT.

Our results should be interpreted in light of the controversies about isolated calf DVT. It is known that venous ultrasonography is less accurate for isolated distal deep venous thrombosis than for proximal deep venous thrombosis [35]. Additionally, the clinical utility of including the calf veins in venous ultrasound is under debate because there is limited data about the natural course of isolated calf DVT and the benefit of anticoagulation [23, 24]. The only randomized, double-blind, placebo-controlled trial comparing low-molecular-weight heparin to placebo for acute symptomatic calf DVT found that low-molecular-weight heparin was not superior to placebo in reducing the risk of proximal extension or venous thromboembolic events, but did increase the risk of bleeding [19]. On the other hand, a non-negligible long-term risk of recurrent venous thromboembolism was found when isolated calf DVT was treated with only a short-term (4–6 weeks) treatment of low-molecular weight heparin, in particular in patients with unprovoked isolated calf DVT or cancer [36].

According to the consensus guideline of the German, Swiss and Austrian Societies for Vascular Medicine [3] an additional advantage of whole-leg ultrasound is to identify differential diagnoses for example Baker’s cyst, muscle fiber tear, aneurysm, hematoma, tumor as the reason for the patient’s symptoms if negative for acute DVT. A recent study demonstrated that alternative diagnoses explaining leg symptoms can be detected on whole-leg ultrasound in two thirds of patients with no evidence of acute DVT [27].

Several limitations of our investigation should be mentioned. This single-center study had a limited cohort size and was performed at a university hospital. This may be a more selected cohort of patients than patients with suspected DVT seen by primary care physicians introducing possible selection bias. No external reference standard is available to confirm findings at ultrasound. Due to the retrospective nature of our investigation, Wells scores and D-Dimer levels were not available for all patients. Also, the precise time from symptom onset was not available in all cases. This may affect the findings on D-Dimer levels, which typically change over the course of acute DVT.

## Conclusion

In summary, isolated calf DVT represents approximately half of DVT cases and has different clinical characteristics than proximal DVT. Venous ultrasound with a limited range from the groin to the knee will miss these cases.

## Data Availability

All materials described in the manuscript, including all relevant raw data, will be freely available from the corresponding author upon reasonable request by any scientist wishing to use them for non-commercial purposes.

## Abbreviations

DVT: Deep venous thrombosis
PE: Pulmonary embolism
